# Editorial: Crosslinking neuropsychiatric symptoms across the continuum of Alzheimer's disease and related dementias

**DOI:** 10.3389/frdem.2024.1498924

**Published:** 2024-11-06

**Authors:** Foteini Christidi, Christos Bakirtzis, Gita A. Pathak, Carolyn Fredericks, Catherine Munro

**Affiliations:** ^1^First Department of Neurology, School of Medicine, National and Kapodistrian University of Athens, Athens, Greece; ^2^Second Department of Psychiatry, School of Medicine, National and Kapodistrian University of Athens, Athens, Greece; ^3^Department of Neurology, School of Medicine, Democritus University of Thrace, Alexandroupolis, Greece; ^4^Computational Neuroimaging Group, Trinity College Dublin, Dublin, Ireland; ^5^2nd Department of Neurology, School of Medicine, Aristotle University of Thessaloniki, Thessaloniki, Greece; ^6^Department of Psychiatry, Yale University School of Medicine, New Haven, CT, United States; ^7^Veteran Affairs Connecticut Healthcare System, West Haven, CT, United States; ^8^Institute for Genomic Health, Icahn School of Medicine at Mount Sinai, New York, NY, United States; ^9^Department of Neurology, Yale University School of Medicine, New Haven, CT, United States; ^10^Brigham and Women's Hospital, Department of Neurology, Boston, MA, United States; ^11^Massachusetts General Hospital, Department of Psychiatry, Boston, MA, United States; ^12^Harvard Medical School, Department of Neurology, Boston, MA, United States

**Keywords:** neuropsychiatric symptoms, Alzheimer's disease, dementias, agitation, Lewy Body Disease, apathy, cognitive behavioral therapies, depression

People with dementia commonly have neuropsychiatric symptoms such as depression, anxiety, apathy, agitation, delusions, hallucinations, and sleep disorders, among other symptoms (Cummings, 2021). There is evidence that ~97% of people with dementia experience at least one neuropsychiatric symptom since the onset of dementia symptoms (Steinberg et al., 2008), and new research suggests neuropsychiatric symptoms may be present even in preclinical and early clinical stages of cognitive impairment (Eikelboom et al., 2021). However, it can be difficult to identify and treat these symptoms across stages of cognitive decline and neurodegeneration (Lyketsos, 2015). In preclinical stages, it can be challenging to parse out whether neuropsychiatric symptoms represent early manifestations of neurodegenerative disorders or are better explained by a primary psychiatric condition (Monastero et al., 2009). In addition, it is still poorly understood how trajectories of neuropsychiatric symptoms progress over time in neurodegenerative disease (Borsje et al., 2015), and whether neuropsychiatric symptoms may have a negative prognostic value for the progression of dementia (Peters et al., 2015).

Our Research Topic aims to highlight studies that focus on better understanding of the relationship between neuropsychiatric symptoms among neurodegenerative diseases. Understanding trajectories of neuropsychiatric symptoms will advance knowledge for earlier identification and treatment in individuals with Alzheimer's Disease and Related Dementias (ADRD). Characterizing molecular mechanisms underlying neuropsychiatric symptoms in ADRD will identify potential biological targets for drug development and repurposing efforts.

Research Topics of interest included the following:

- *Demographic effects on ADRD with potential implication on the study of neuropsychiatric symptoms in ADRD*

Stanley et al. applied logistic regression analysis to determine gender-related differences in demographic and pharmacological factors in a large clinical sample of 9,290 individuals with Alzheimer's disease, 6,039 individuals with Vascular Dementia, and 412 individuals with mixed dementias: vascular-Alzheimer's disease. They found gender differences and similarities in age, medical history (e.g., tobacco use), and medication of individuals with Alzheimer's dementia, vascular dementia, and mixed dementias vascular-Alzheimer's disease, providing evidence for the necessity to investigate gender-related effects in ADRDs, including those probably associated with neuropsychiatric symptoms.

- *Valid psychometric tools to identify and determine meaningful changes of specific neuropsychiatric symptoms over time in ADRD*

Meunier et al. conducted a *post-hoc* analysis of data from the brexpiprazole clinical program to determine a meaningful within-patient change (MWPC) threshold for the Cohen-Mansfield Agitation Inventory (CMAI) that quantifies the frequency of agitation behaviors in elderly persons. By analyzing data from 898 patients, the authors highlighted that a MWPC threshold of −20 points for the CMAI total score could predict dementia due to Alzheimer's disease in patients with agitation.

- *Epidemiological metrics of specific neuropsychiatric symptoms and their association with functional impairment in ADRD*

Zhu et al. examined the prevalence and persistence of apathy in 676 well-characterized patients with Lewy Body Disease (LBD) and estimated the effect of apathy on functional impairment over time. They found that >50% of patients showed apathy both at baseline and follow-up evaluation, whereas apathy was strongly associated with greater functional impairment at baseline and faster rate of decline over time. Of note, these effects were beyond the effects on functional impairment from cognitive scores and parkinsonism, underscoring the importance of apathy evaluation in LBD.

- *Genetic basis and causal relationships between education and neuropsychiatric symptoms with potential implications in the study of ADRD*

Chen et al. explored the shared genetic basis and causal relationships between educational attainment (EA) and neuropsychiatric disorders (NPDs) using the high-definition likelihood method, cross phenotype association study, transcriptome-wide association study and bidirectional Mendelian randomization with summary-level data for EA (*N* = 293,723) and NPDs (*N* range = 9,725 to 455,258). The authors identified significant genetic correlations between EA and 12 NPDs. Overall, the study revealed (a) 37 independent loci shared between EA and NPDs, (b) enriched shared genes in brain tissue, especially in the cerebellum, and (c) the role of three regions (6q16.1, 3p21.31, and 17q21.31) for the shared causes between EA and NPDs. The study provided evidence of potential biological pathways underlying both EA and NPDs.

- *Non-pharmacological treatment of neuropsychiatric symptoms in ADRD*

Lastly, in a systematic review and meta-analysis study, González-Martín et al. focused on the effects of cognitive behavioral therapies (CBT) on depression in older adults with Alzheimer's disease. Despite the heterogeneity in the protocols used across the 11 studies included, the authors reported that psychosocial CBT is effective in improving depression in individuals with Alzheimer's disease.

In conclusion, this Research Topic of studies promoted discussion on neuropsychiatric symptoms in ADRD, highlighting the role of demographic and genetic factors and providing low-cost measures to effectively diagnose and treat ADRD-associated neuropsychiatric symptoms.
